# Sulfur‐Decorated Ni−N−C Catalyst for Electrocatalytic CO_2_ Reduction with Near 100 % CO Selectivity

**DOI:** 10.1002/cssc.202200870

**Published:** 2022-09-01

**Authors:** Song Lu, Yang Zhang, Mohamed F. Mady, Obinna Egwu Eleri, Wakshum Mekonnen Tucho, Michal Mazur, Ang Li, Fengliu Lou, Minfen Gu, Zhixin Yu

**Affiliations:** ^1^ Department of Energy and Petroleum Engineering University of Stavanger 4036 Stavanger Norway; ^2^ Beyonder AS Kanalsletta 2 4033 Stavanger Norway; ^3^ Department of Chemistry Bioscience and Environmental Technology University of Stavanger 4036 Stavanger Norway; ^4^ Department of Mechanical and Structural Engineering and Material Science University of Stavanger 4036 Stavanger Norway; ^5^ Department of Physical and Macromolecular Chemistry Faculty of Science Charles University Hlavova 8 12843 Prague 2 Czech Republic; ^6^ Center for Analysis and Testing Nanjing Normal University 210023 Nanjing P. R. China

**Keywords:** CO_2_ reduction, density functional theory, doping, electrocatalysis, nickel

## Abstract

Developing highly efficient electrocatalysts for electrochemical CO_2_ reduction (ECR) to value‐added products is important for CO_2_ conversion and utilization technologies. In this work, a sulfur‐doped Ni−N−C catalyst is fabricated through a facile ion‐adsorption and pyrolysis treatment. The resulting Ni−NS−C catalyst exhibits higher activity in ECR to CO than S‐free Ni−N−C, yielding a current density of 20.5 mA cm^−2^ under −0.80 V versus a reversible hydrogen electrode (vs. RHE) and a maximum CO faradaic efficiency of nearly 100 %. It also displays excellent stability with negligible activity decay after electrocatalysis for 19 h. A combination of experimental investigations and DFT calculations demonstrates that the high activity and selectivity of ECR to CO is due to a synergistic effect of the S and Ni−N_*X*_ moieties. This work provides insights for the design and synthesis of nonmetal atom‐decorated M−N−C‐based ECR electrocatalysts.

## Graphical abstract

**Approaching perfection**: A sulfur‐doped Ni−N−C catalyst (Ni−NS−C) is synthesized by a facile ion‐adsorption and pyrolysis treatment method, which yields a current density of 20.5 mA cm^−2^ under −0.80 V vs. RHE and maximum CO faradaic efficiency of nearly 100 % during electrochemical CO_2_ reduction.

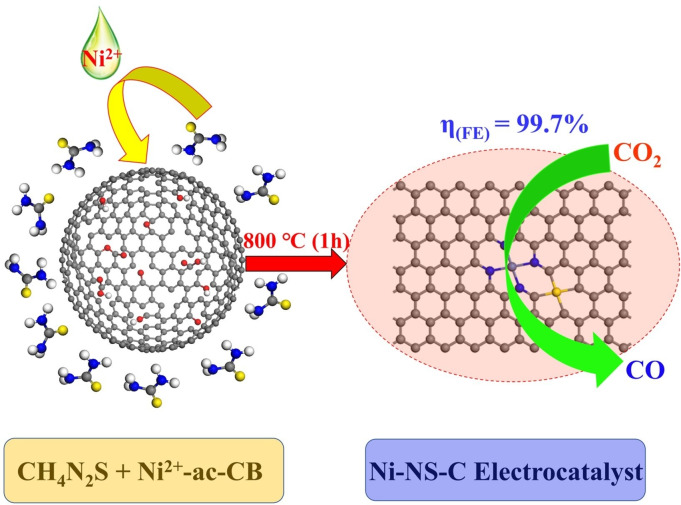

## Introduction

About 500 gigatons of carbon dioxide will be generated from the combustion of fossil fuels in the next half century.^[1]^ Excessive CO_2_ emissions have aroused environmental issues such as global warming and erratic weather.[^2^, ^3^] To alleviate these adverse effects, developing efficient carbon capture, utilization and storage (CCUS) technologies is of paramount importance. In recent years, electrochemical CO_2_ reduction (ECR), potentially powered by renewable electricity sources, has received extensive attention. ECR can convert CO_2_ to value‐added products such as C_1_ (e. g., CO, HCOOH, CH_4_), C_2_ (e. g., C_2_H_4_, C_2_H_5_OH), and C_3_ (e. g., C_3_H_7_OH) under ambient temperature and pressure.[^4^, ^5^, ^6^, ^7^] In particular, it has been shown that ECR to CO by a two‐electron reduction process is the most promising for commercialization, owing to high selectivity and low energy requirement.[^8^, ^9^] Moreover, CO as an important chemical feedstock plays a vital role in various industries.^[10]^ Even though much progress has been made in ECR to CO, it is still far from commercial application for reasons such as unsatisfactory activity, poor stability, and high cost. In addition, the competing hydrogen evolution reaction (HER) severely suppresses the CO selectivity in aqueous solutions.^[11]^ Therefore, it is desirable to exploit CO_2_‐to‐CO electrocatalysts with excellent activity and high selectivity.

Among CO_2_‐to‐CO electrocatalysts explored, nitrogen‐doped carbon‐based material‐supported transition metal atoms (M−N−C) exhibit great potential for CO generation.[^12^, ^13^, ^14^, ^15^, ^16^, ^17^] Carbon‐based materials are utilized, owing to good electrical conductivity, which benefits electron transportation. Among these materials, carbon black (CB) not only exhibits merits such as large surface area and confinement of metal atoms, but also facilitates CO_2_ diffusion across the gas diffusion barrier and accelerate the reaction rate.[^18^, ^19^, ^20^] Therefore, CB serves as substrate that could cost‐effectively improve the atomic dispersion of M and the activity of ECR to CO. The M−N_*X*_ moieties in these catalysts are usually regarded as active center where *X* denotes the coordination number of metal atoms. In addition, the M−N_*X*_ moiety with a planar coordination structure has been confirmed to stabilize the metal atoms effectively.^[21]^ More interestingly, nonprecious metals, such as Fe, Co, Ni, or Mn, in M−N_*X*_ system could achieve identical and even better performance than noble metal‐based electrocatalysts. For instance, isolated Fe−N_4_ structure on carbon materials could lower the energy barrier for the formation of *COOH intermediate during ECR to CO, thus enhancing the CO selectivity with a faradaic efficiency [FE(CO)] up to 93 %.^[22]^ Co atom dispersed on N‐doped porous carbon sphere was found to have >90 % FE(CO) under potentials between −0.57 and −0.88 V versus the reversible hydrogen electrode (vs. RHE). Spectroscopic studies and density functional theory (DFT) calculations revealed that Co−N_5_ site serves as the active center for boosting CO_2_ activation and facilitating the formation of *COOH as well as CO desorption.^[23]^ Ni−N_4_ moiety confined in Ni porphyrin‐based framework displayed maximum FE(CO) of 97 % at −0.90 V (vs. RHE) and good stability. Theoretical simulation demonstrated that Ni−N_4_ center could decrease the energy barrier for *COOH intermediate generation.^[24]^ Mn−N_3_ embedded into graphitic carbon nitride shown 98.8 % of FE(CO) under a low overpotential of 0.44 V, and the outstanding activity was attributed to Mn−N_3_ moiety promoting the formation of the key intermediate *COOH.^[25]^ Therefore, it can be concluded that the superior CO_2_‐to‐CO performance on these non‐precious metal‐based M−N−C electrocatalysts can be mainly attributed to the proper interaction between M−N_*X*_ center and intermediates.

Recently, studies disclosed that the intrinsic activity of M−N−C could be further enhanced by introducing other heteroatoms. For instance, Zhang et al. reported that extra chlorine atoms (Cl) coordinated Mn−N_4_−C could change the electron transfer of Mn atom, boosting *COOH adsorption and CO desorption.^[26]^ As a result, the electrocatalyst exhibited a FE(CO) of 97 % and a high current density of about 10 mA cm^−2^ under a low overpotential of 0.49 V. Pan et al. reported that S atoms could lift the Fermi energy of Fe3d state and increase the charge density of Fe atoms.^[27]^ The inherent electrocatalytic activity and selectivity for CO_2_‐to‐CO were modulated by S atoms, resulting in improved interaction strength between Fe atom and intermediates. This unique structure and electronic properties endowed the Fe−NS−C electrocatalyst with a FE(CO) of 98 % under an overpotential of 0.49 V. Indeed, the local electronic structures of host atoms could be altered by doping heteroatoms, further influencing catalytic properties.[^28^, ^29^, ^30^] Thus, it can be anticipated that the incorporation of proper heteroatoms into carbon sites close to the M−N_*X*_ center could tune the electronic structure of metal atoms, which would effectively promote the activation of CO_2_ and modify the binding strength of intermediates during the ECR process. Specifically, incorporating S atom into host materials has been regarded as an effective strategy to improve the activity of various catalytic reactions.[^31^, ^32^, ^33^]

Ni−N−C electrocatalyst have been reported to exhibit good CO_2_‐to‐CO activity. However, there is limited study on S modified Ni−N−C system to regulate the ECR activity for CO_2_‐to‐CO. In this study, we synthesized N,S‐codoped CB incorporating Ni atoms by facile ion‐adsorption and subsequent pyrolysis treatment. The Ni−NS−C catalyst exhibited a very high conversion efficiency of 99.7 % to CO with a high total current density of 20.5 mA cm^−2^ under −0.80 V (vs. RHE), outperforming S‐free Ni−N−C electrocatalyst and other ECR catalysts reported in literature. It also displayed excellent stability without activity decay after electrocatalysis for 19 h. First‐principles computation was carried out to investigate the effect of the S atom decoration on the electronic structures of Ni atoms, catalytic mechanisms, and activity toward ECR as well as HER.

## Results and Discussion

### Electrocatalysts characterization

**Figure 1:**
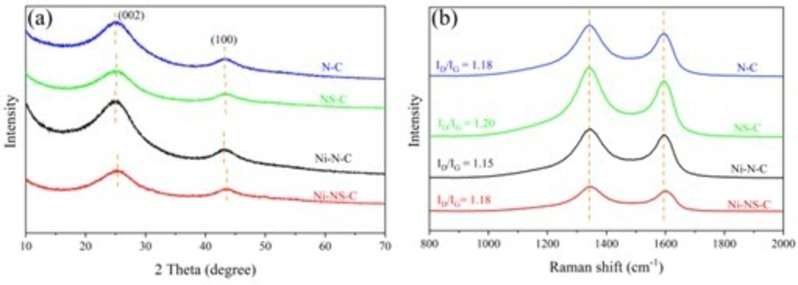
(a) XRD patterns and (b) Raman spectra of N−C, NS−C, Ni−N−C, and Ni−NS−C catalysts.

The structures of N−C, NS−C, Ni−N−C, and Ni−NS−C catalysts were firstly characterized by X‐ray diffraction (XRD). As shown in Figure 1a, the catalysts exhibit similar XRD patterns with two broad diffraction peaks at around 25.1° and 43.2°, corresponding to the (002) and (100) planes of carbon. It is worth noting that both peaks of NS−C and Ni−NS−C catalysts show low crystallinity and slight right‐shift compared with the N−C and Ni−N−C catalysts, which can be explained by lattice contraction and the formation of C vacancy after the introduction of S atoms with larger radius.[^34^, ^35^] Furthermore, no peaks assignable to metallic Ni or its compounds were observable.

Raman spectra of the four catalysts exhibit two vibrational bands located around 1343 cm^−1^ (D band) and 1594 cm^−1^ (G band; Figure 1b), which are ascribed to the defect and graphitic sp^2^ carbon.^[36]^ The intensity ratios of D and G band (*I*_D_/*I*_G_) of N−C, NS−C, Ni−N−C, and Ni−NS−C catalysts were also calculated as marked in Figure 1b. It can be observed that the introduction of S atom into the N−C catalysts induces more defects, consistent with XRD study. However, incorporating Ni atoms decreases the *I*_D_/*I*_G_ value, indicating that Ni atoms are embedded into C vacancies. Compared with the Ni−N−C catalyst, the position of the G band of Ni−NS−C catalysts shows a slight upshift owing to doping of nonmetal S atoms into carbon‐based materials.[^37^, ^38^, ^39^]

**Figure 2:**
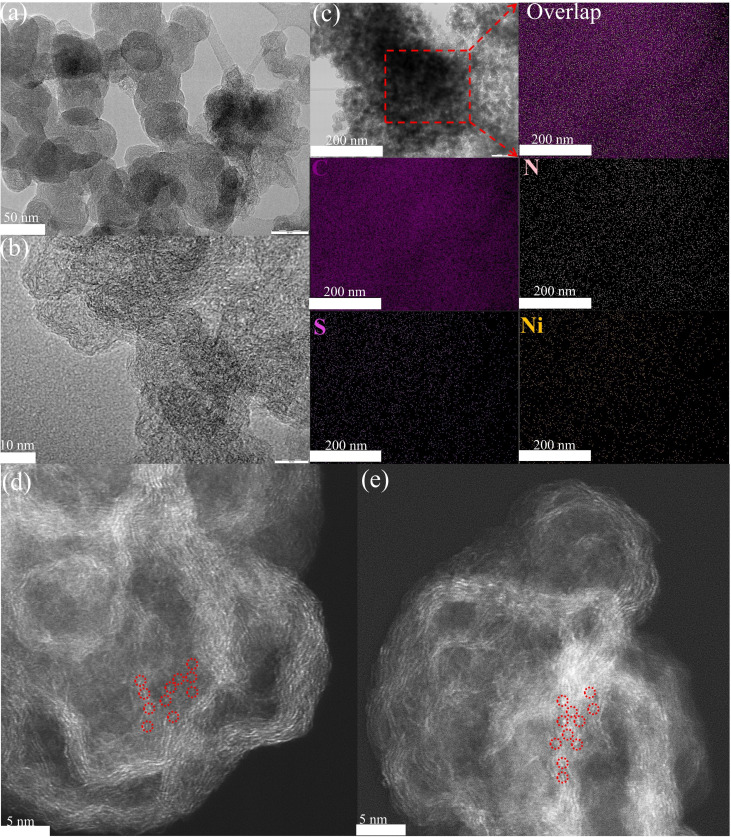
(a) TEM, (b) HRTEM, and (c) C, N, S, and Ni EDX mapping images of Ni−NS−C catalyst; (d, e) HAADF‐STEM images for Ni−N−C and Ni−NS−C catalysts, where single Ni atoms are highlighted in red circles.

Transmission electron microscopy (TEM) was used to investigate the microscopic morphology of the Ni−NS−C catalyst. Carbon nanospheres with a diameter of 50 nm were observed (Figure 2a). The high resolution transmission electron microscopy (HRTEM) images displays distorted short‐range graphic stripes with winkle and interlaces (Figure 2b), indicating defective carbon structure. No distinct nanoparticles or clusters were observed, implying that the Ni atoms are likely to present in the form of single atoms. Moreover, energy‐dispersive X‐ray spectroscopy (EDX) elemental mapping images clearly demonstrate that Ni, N, S and C species distribute uniformly over the Ni−NS−C catalyst (Figure 2c). Furthermore, the aberration‐corrected high‐angle annular dark‐field scanning transmission electron microscopy (HAADF‐STEM) shows the well‐dispersed single Ni atoms as bright spots for the Ni−N−C and Ni−NS−C catalysts, which are highlighted by red circles in Figure 2d and e.

**Figure 3:**
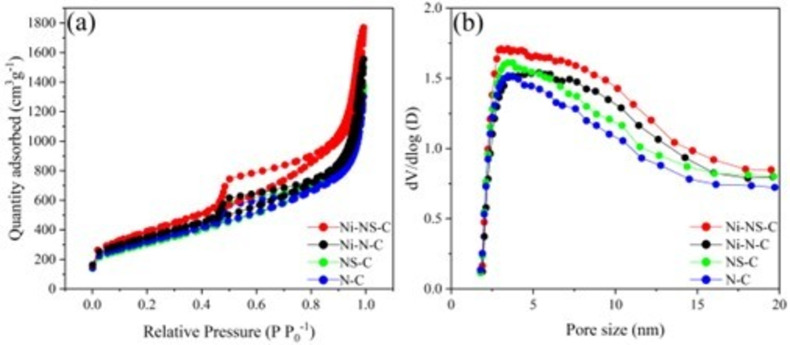
(a) N_2_ adsorption–desorption isotherms and (b) pore size distributions of N−C, NS−C, Ni−N−C, and Ni−NS−C catalysts.

The surface area and pore structure of the catalysts were determined by N_2_ adsorption‐desorption measurements. As depicted in Figure 3a, the isotherms of the four electrocatalysts displayed sharp adsorption under relative pressures greater than 0.40 accompanied by an obvious hysteresis loop, which is indicative of dominant mesopores and is further corroborated by the pore size distributions (Figure 3b). The specific surface area and pore volume are summarized in Table S1 (see the Supporting Information). It can be found that the total pore volume increased from 1.26 to 1.39 m^3^ g^−1^ after doping S to the N−C structure, and increased further after introducing Ni atoms. The catalysts show quite close but very high surface areas in the range of 1073 to 1275 m^2^ g^−1^, which also increase with the doping of S and Ni atoms. The high surface area and pore volume are beneficial for the dispersion of Ni atoms and the access of reactants to the active centers. As expected, the pore size distribution curve also shows more larger pores with the introduction of S and Ni atoms (Figure 3b).

**Figure 4:**
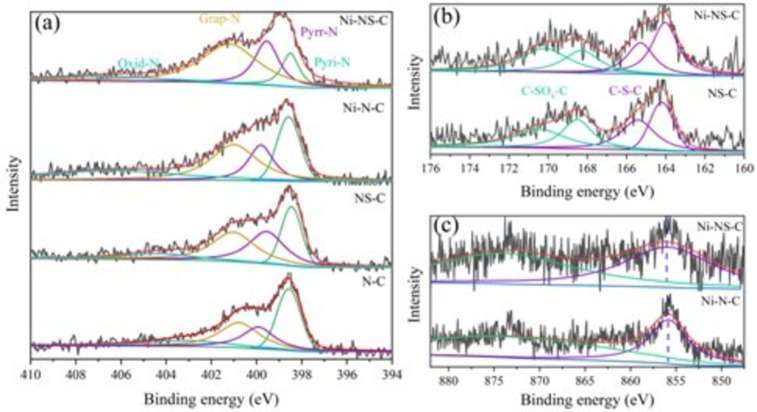
High‐resolution XPS spectra: (a) N1s for N−C, NS−C, Ni−N−C, and Ni−NS−C catalysts; (b) S2p for NS−C and Ni−NS−C catalysts; (c) Ni2p for Ni−N−C and Ni−NS−C catalysts.

X‐ray photoelectron spectroscopy (XPS) was employed to characterize the chemical state and surface concentration of the elements. The high‐resolution N1s spectra of the Ni−NS−C catalyst can be fitted into four peaks cantered at 397.9, 399.7, 400.5 and 403.1 eV (Figure 4a), which can be assigned to pyridinic N (Pyri‐N), pyrrolic (Pyrr‐N), graphitic N (Grap‐N) and oxidized N (Oxid‐N), respectively.[^40^, ^41^, ^42^, ^43^] The existence of these types of N could promote the electrocatalytic activity.^[44]^ In addition, N atom concentrations are 4.43, 4.19, 4.11 and 4.18 at.% for N−C, NS−C, Ni−N−C, and Ni−NS−C (Table S1). High‐resolution S2p spectra of Ni−NS−C catalyst (Figure 4b) at lower binding energy can be ascribed as C−S−C (2p_3/2_ at 164.1 and 2p_1/2_ at 165.3 eV) and C−SO_*x*_−C (167.6 and 168.8 eV).[^34^, ^45^] The S content was estimated to be 0.37 and 0.42 at.% for the NS−C and Ni−NS−C catalyst, respectively. It was also observed that the percentages of N in these two catalysts remain almost unchanged, implying that introducing S atoms had little effects on the bonding patterns of N atoms. In the high‐resolution Ni2p spectrum (Figure 4c), the Ni2p_3/2_ binding energies for Ni−NS−C and Ni−N−C catalyst are 855.90 and 855.66 eV, higher than that of Ni^0^ (852.5–853.0 eV) but lower than that of Ni^2+^ (856 eV),[^46^, ^47^] indicating that Ni species are likely to keep as ionic Ni^*δ*+^ (0<*δ*<2). Therefore, these results further demonstrated the existence of single Ni atoms on the surface of Ni−NS−C and Ni−N−C catalysts.^[48]^ Moreover, the peak of Ni2p_3/2_ shifted slightly towards higher binging energy after incorporating S atoms, indicating that S could influence the electronic structure of Ni. The Ni contents in Ni−N−C and Ni−NS−C catalysts are 0.50 and 0.48 at.%, demonstrating that S atom doping has little effect on surface Ni atom distribution.

### Electrocatalytic activity test

**Figure 5:**
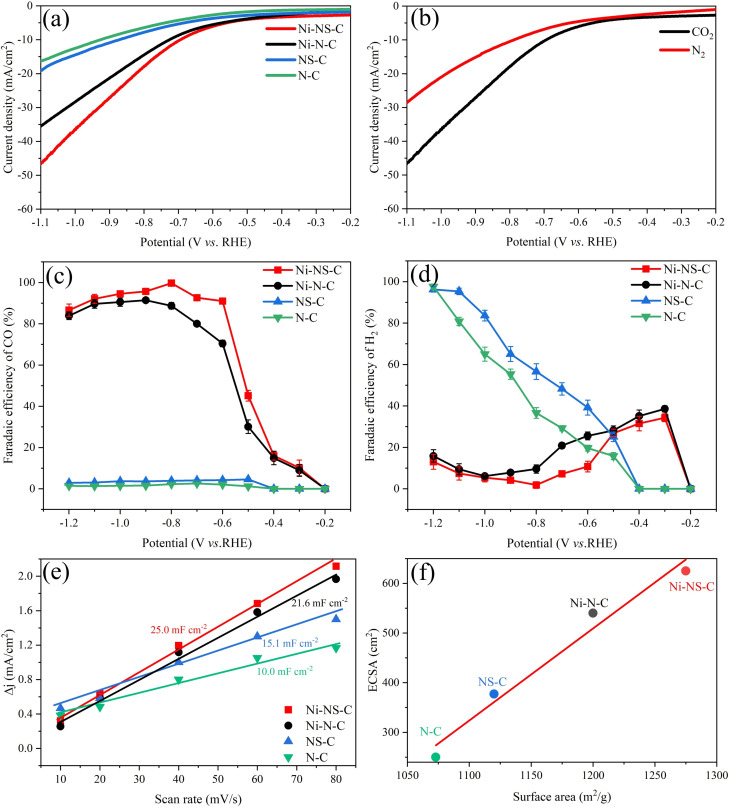
(a) LSV curves of N−C, NS−C, Ni−N−C, and Ni−NS−C catalysts under CO_2_‐saturated 0.5 m KHCO_3_ solution at a scan rate of 10 mV s^−1^; (b) LSV curves of Ni−NS−C catalyst under N_2_‐ and CO_2_‐saturated 0.5 m KHCO_3_ solution at a scan rate of 10 mV s^−1^; (c) faradaic efficiencies of CO generation and (d) faradaic efficiencies of H_2_ generation on N−C, NS−C, Ni−N−C, and Ni−NS−C catalysts; (e) Charging current density differences against scan rates over N−C, NS−C, Ni−N−C, and Ni−NS−C catalysts; (f) relationship between double layer capacity and BET surface area of the N−C, NS−C, Ni−N−C, and Ni−NS−C catalysts.

The ECR performances of catalysts were evaluated in a membrane‐separated two chambers H‐type cell with a standard three‐electrode system immersed in 0.5 m KHCO_3_ electrolyte. Under CO_2_‐saturated electrolyte, the Linear sweep voltammetry (LSV) of N−C and NS−C catalysts show small current density (Figure 5a), exhibiting low electrocatalytic activity. Conversely, the Ni−NS−C and Ni−N−C catalyst display large current density thus high electrocatalytic activity. In addition, the doping of S atoms could boost the current density for both N−C and Ni−N−C, confirming the role of S atom in the activity enhancement. In the presence of CO_2_, the current density of Ni−NS−C increases faster in comparison with the reaction under N_2_ atmosphere (Figure 5b), suggesting the enhanced current density from ECR. The results demonstrated that introducing Ni−N_*X*_ moiety into carbon‐based materials could indeed improve its electrocatalytic performance. Meanwhile, the highest current density observed for the Ni−NS−C catalyst could be fairly attributed to the synergistic effect of Ni−N_*X*_ moiety and S doping.

To quantify the product selectivity, the ECR test was conducted in constant potentiostatic electrocatalysis under different potentials. H_2_ and CO were detected as the only reduction products under the potential window of −0.20 V to −1.20 V (vs. RHE). No other gaseous products were detected by GC, because CO, as a two‐electron product, is more easily generated than other four‐, six‐, or eight‐electron products such as CH_3_OH and CH_4_, consistent with previous studies.[^49^, ^50^] Moreover, no liquid products were produced, as evidenced by ^1^H NMR analysis (see the Supporting Information, Figure S1). As shown in Figure 5c, the FE(CO) over the four catalysts showed first increase then decrease with the decrease of applied potential. However, the FE(CO) of Ni−N−C and Ni−NS−C catalysts are always higher than the N−C and NS−C catalysts under the same applied potential, indicating that Ni−N_*X*_ are the true active centers. Meanwhile, the Ni−NS−C catalyst exhibits larger FE(CO) than Ni−N−C over the entire potential range, demonstrating that S atom could effectively enhance the ECR performance of the Ni−N_*X*_ moiety. The competing HER performance for the four catalysts were also compared, as displayed in Figure 5d. The FE(H_2_) of Ni−NS−C catalyst is always lower than that of Ni−N−C catalyst, indicating that the HER performance of the Ni−N−C catalyst was suppressed after the doping of S atoms. In contrast, the FE(H_2_) of N−C is lower than that of NS−C, suggesting that S atom could promote HER ability of N−C in the absence of Ni. Therefore, it can be deduced that more protons are involved in ECR compared to HER after the introduction of Ni and S atoms, and there is a synergistic effect between Ni and S. As a result, the Ni−NS−C catalyst exhibits high FE(CO) over 90 % in a broad potential range of −0.60 to −1.10 V (vs. RHE), and the maximum FE(CO) is as high as 99.7 % at a potential of −0.80 V (vs. RHE) with a total current density of 20.5 mA cm^−2^.

The increase of electrochemical active surface areas (ECSA) also contributes to the excellent activity towards ECR. ECSA can be directly estimated by measuring double layer (D–L) capacitance (Figure 5e and Figure S2). The Ni−NS−C and Ni−N−C catalysts exhibit 25.0 mF cm^−2^ and 21.6 mF cm^−2^, which are larger than that of the NS−C (15.1 mF cm^−2^) and N−C catalysts (10.0 mF cm^−2^). Consequently, the corresponding ECSA for Ni−NS−C, Ni−N−C, NS−C and N−C catalysts were 625, 540, 377 and 250 cm^2^_ECSA_, demonstrating that Ni and S atoms could effectively increase the ESCA (Table S1). It can also be found that there is a positive correlation between ECSA and BET surface area (Figure 5f), validating that larger surface area could expose more active sites.

CO_2_ adsorption on catalyst surface plays an important role in ECR. Therefore, we conducted temperature‐programmed CO_2_ desorption (CO_2_‐TPD) to investigate the effect of S dopant on their CO_2_ adsorption ability. It turns out that Ni−NS−C shows slightly stronger CO_2_ adsorption than that of Ni−N−C, which could boost the ECR performance (Figure S3). To further elucidate the effect of S atom on the reaction kinetics, the Tafel slope and electrochemical impedance spectroscopy (EIS) were conducted.

**Figure 6:**
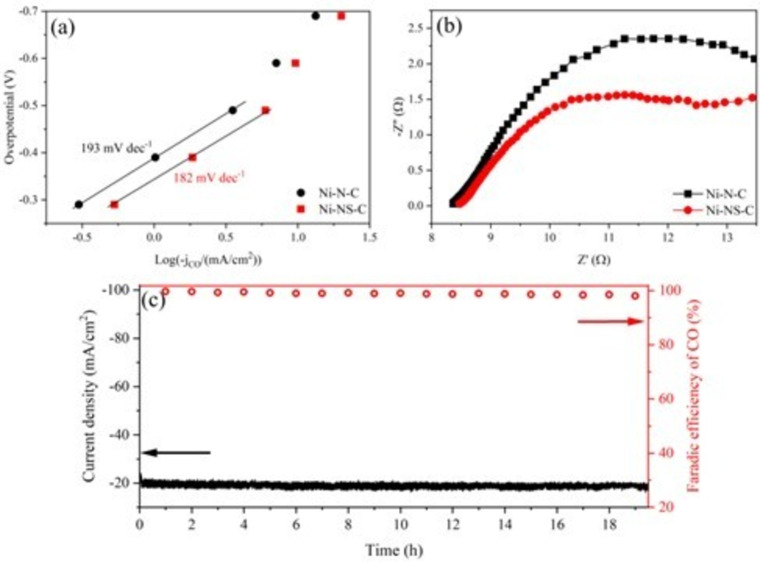
(a) Tafel plots for CO generation on Ni−N−C and Ni−NS−C catalysts; (b) EIS complex‐plane plot for Ni−NS−C and Ni−N−C catalysts in 0.5 m KHCO_3_ at potential of −0.8 V (vs. RHE); (c) Long‐term electrocatalysis on Ni−NS−C catalyst in 0.5 m KHCO_3_ at −0.8 V (vs. RHE).

As shown in Figure 6a, the Ni−NS−C catalyst exhibits a lower Tafel slope of 182 mV dec^−1^ than Ni−N−C of 193 mV dec^−1^, suggesting that the introduction of S atom could improve the reaction kinetics. The Tafel slopes of these two samples are close to the theoretical value, revealing that CO_2_‐to‐CO on these two catalysts proceed via the same mechanism that CO_2_ accepting proton‐electron pairs to form *COOH intermediate is the potential determining step (PDS).^[51]^ The EIS test was further conducted at −0.80 V (vs. RHE) and the corresponding complex‐plane plot of Ni−N−C and Ni−NS−C catalysts is shown in Figure 6b. The Ni−NS−C catalyst exhibited a smaller charge transfer resistance (*R*_ct_), demonstrating that incorporating S atom could accelerateelectron exchange between the catalysts and reactants. In addition, the electrochemical stability of Ni−NS−C was evaluated by chronoamperometric electrolysis under the potential of −0.80 V (vs. RHE) with the largest FE(CO). As shown in Figure 6c, there is a fast drop in current density during the initial 20 min, which can be attributed to unbalanced CO_2_ adsorption on the catalyst. After that, the current density and FE (CO) is highly stable with negligible decay. The current density is still around 20.5 mA cm^−2^ and the FE (CO) is about 98 % after electrocatalysis reaction for >19 h. We also studied the structure and element information of the spent Ni−NS−C catalyst by TEM, EDX mapping and XPS (Figures S4 and S5; summarized in Table S1). It can be found that there is no obvious change in morphology and element content after ECR reaction, demonstrating excellent stability of the Ni−NS−C catalyst. Very recent reports on nonmetal‐decorated M−N−C catalysts are summarized in Table S2. Remarkably, the Ni−NS−C catalyst in this study outperformed most of the catalysts in terms of applied potential, FE(CO) and stability.

### DFT simulations

**Figure 7:**
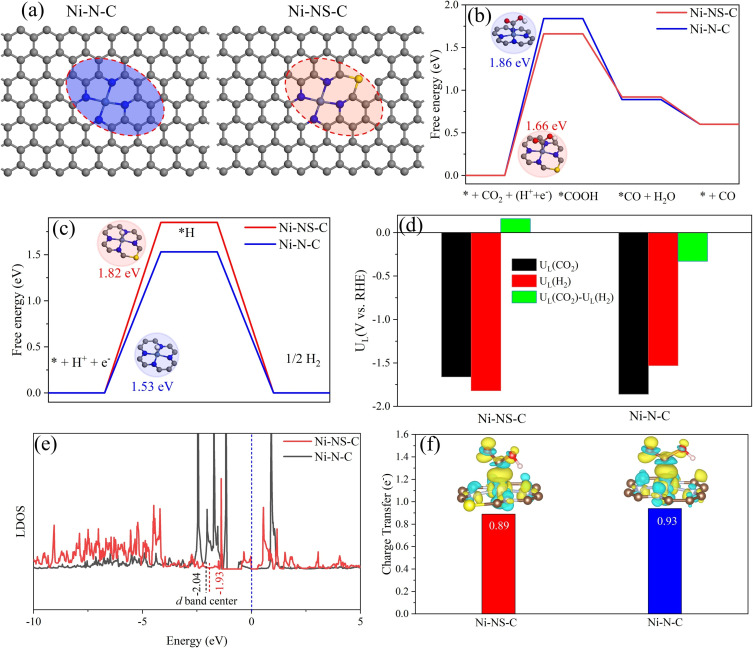
(a) Atomic structure of proposed Ni−N−C and Ni−NS−C catalysts; (b) free energy diagrams for ECR to CO; (c) free energy diagrams for HER; (d) difference between the limiting potentials for ECR and HER; (e) location of d band center; (f) charge density difference after *COOH adsorption on Ni−N−C and Ni−NS−C catalysts, the isosurface value is set to be 0.003 e Bohr^−3^.

DFT calculations were performed to gain insight into the electrocatalytic reaction mechanisms of ECR and HER on Ni−N−C and Ni−NS−C catalysts based on the CHE model. As Ni atoms are more likely to form stable Ni−N_4_ moiety in carbon‐based materials, the Ni−N_4_ structure was created in carbon matrix as active center in Ni−N_4_−C catalyst.[^13^, ^52^, ^53^, ^54^] It is worth noting that similar method for synthesizing Fe−N_4_−C catalyst has been reported recently.^[55]^ The good ECR activity of Ni‐decorated nitrogen‐doped carbon catalysts was previously attributed to single Ni atoms instead of Ni nanoparticles.^[48]^ XRD indicated that the introduction of S atoms possibly induced the formation of C atom vacancies because the radius of S atom is larger than that of C atom (Figure 1a). To exclude the possibility of S directly substituting C atom without breaking TM−N_4_, we also built a configuration as shown in Figure S6, where S directly substituted C atom but TM−N_4_ was maintained. Compared to the pristine S free structure, the CO desorption ability will be quite weak in this structure because of high free energy change of 1.46 eV, whereas the HER ability was strengthened owing to low free energy of −0.53 eV. These results are not consistent with the experimental data. Therefore, S atom embedded into C vacancy close to Ni site (Ni−NS−C) as model was proposed to investigate the effect of S atoms on the ECR performance of the Ni−N_4_−C catalyst (Figure 7a). The adsorption energy of CO_2_ on the Ni−N−C and Ni−NS−C catalysts were calculated to be −0.28 and −0.39 eV, further demonstrating that S dopant could promote CO_2_ adsorption, consistent with the CO_2_‐TPD results. The adsorbed *COOH, *CO and *H intermediates were considered in ECR and HER. As shown in Figure 7b, the Gibbs free energy change (Δ*G*) diagram of CO_2_‐to‐CO over these two catalysts suggests that the first proton‐electron pair to generate *COOH is the PDS, consistent with the experimental results. The Ni−N−C catalyst shows ΔG_*COOH_ of 1.86 eV, which is higher than that of the Ni−NS−C catalyst at 1.66 eV, confirming that S atom could effectively decrease the CO_2_ activation barrier. This is also consistent with the experimental result that introducing S atoms could enhance FE(CO) under all applied potentials. The calculated adsorption energy of CO over the Ni−N−C catalyst was reduced by 0.06 eV after the incorporation of S atom, demonstrating the increase of CO desorption ability. The HER activities over the two catalysts were compared by calculating the Gibbs free energy of *H (ΔG_*H_; Figure 7c). Notably, the ΔG_*H_ of Ni−NS−C catalyst increases from 1.53 to 1.82 eV, showing that S atom could effectively suppress HER when Ni is present, which again agrees with the experimental observation that FE(H_2_) of the Ni−N−C catalyst decreased after doping S (Figure 5d).

It has been well established that the difference between the limiting potentials of ECR and HER [*U*_L_(CO_2_)−*U*_L_(H_2_)] could be a reasonable descriptor for ECR selectivity. A more positive *U*_L_(CO_2_)−*U*_L_(H_2_) indicates a higher selectivity towards ECR. As displayed in Figure 7d, the Ni−NS−C catalyst shows a positive value of *U*_L_(CO_2_)−*U*_L_(H_2_), which is negative for the Ni−N−C catalyst, demonstrating increased ECR selectivity after incorporating S atoms. Furthermore, the electronic structure of Ni−NS−C and Ni−N−C catalysts were compared. The d band center of Ni−N−C catalyst moved towards the Fermi level after introducing the S atom, and the Ni3d state of Ni−NS−C catalyst crossed the Fermi level (Figure 7e), thereby improving the intrinsic activity of 3d electrons. When the *COOH intermediate was adsorbed on the catalysts, Ni atom (Ni−NS−C) lost smaller charge (0.89e^−^; Figure 7f), accounting for moderate interaction. The results demonstrated that the synergistic effect of the Ni and S atom boosts the ECR performance where the Ni atoms sever as the active center and S atom plays a role in the modification of electronic properties.

## Conclusion

We have developed a facile method for the synthesis of a S‐ and Ni‐doped N−C system as an electrocatalyst for CO_2_ reduction to CO. The Ni−NS−C catalyst can selectively reduce CO_2_ to CO with high a FE(CO) of over 90 % in a broad potential range of −0.60 to −1.10 V (vs. RHE). The maximum FE(CO) reaches 99.7 % at −0.80 V (vs. RHE) with a total current density of 20.5 mA cm^−2^. It also exhibits excellent stability for 19 h electrocatalysis without apparent activity decay. Experimental results demonstrated that Ni atoms served as active sites for ECR to CO, whereas S atoms could increase its activity further. Moreover, theoretical calculations disclosed that doping S atoms could decrease and increase the free energy barrier for the formation of *COOH and *H, respectively. Meanwhile, S atoms could lift the d band center of Ni atoms and induce the Ni3d state crossing the Fermi energy level. As a result, the performance of ECR to CO on Ni−NS−C was improved, whereas the hydrogen evolution reaction (HER) was suppressed, contributing to overall higher ECR selectivity. Therefore, the excellent ECR performance of the Ni−NS−C catalyst can be attributed to the synergistic effect of the Ni−N_*X*_ moiety and S dopant.

## Experimental Section

### Chemicals

All chemicals were analytical grade and used without further purification. Ketjenblack EC‐600 JD (CB) was purchased from AkzoNobel. Nickel hexahydrate nitrate (Ni(NO_3_)_2_ ⋅ 6H_2_O, 98 %) was obtained from Sigma‐Aldrich. Urea (CH_4_N_2_O, 99 %), thiourea (CH_4_N_2_S, 99 %) and nitric acid (HNO_3_, 65 wt%) were from VMR chemicals. Potassium bicarbonate (KHCO_3_, 98 %), Nafion D‐521 dispersion (5 % w/w in water and 1‐propanol) and Nafion‐117 ionic exchange membrane were purchased from Alfa Aesar. Deionized (DI) water was produced by a Milli‐Q (18.2 MΩ cm) system.

### Electrocatalyst preparation

The CB was firstly activated by concentrated HNO_3_ solution to increase surface defects and oxygen‐containing groups. Typically, 4 g CB was dispersed in 100 ml of HNO_3_ solution followed by refluxing at 100 °C for 8 h with vigorously stirring. Subsequently, the suspension was washed with DI water several times until neutral pH and separated by vacuum filtration. Activated CB was obtained after drying at 120 °C in a vacuum oven for 24 h.

In a typical synthesis of Ni^2+^ adsorbed on CB (Ni^2+^‐CB), 1 g activated CB was dispersed in 400 mL DI water under sonication for 2 h. The Ni^2+^ solution (3 mg mL^−1^) was prepared by dissolving 240 mg Ni (NO_3_)_2_ ⋅ 6H_2_O in 80 mL DI water. Thereafter, 40 mL Ni^2+^ solution was added dropwise into the CB solution and kept under vigorous stirring for 12 h. The products were collected by vacuum filtration. After drying at 120 °C in a vacuum oven for 24 h, Ni‐CB was obtained.

The Ni−NS−C catalyst was synthesized according to a modified method by Zheng et al.^[18]^ Typically, 0.5 g Ni‐CB and with 6.0 g thiourea was mixed and grinded to obtain fine powders. The powder was then transferred into a covered crucible and heated to 800 °C at a heating rate of 3 °C min^−1^ under 10 mL min^−1^ Ar flow and kept at 800 °C for 1 h. For comparison, the Ni−N−C catalyst was synthesized by replacing thiourea with urea. Metal free N−C and NS−C catalysts were also prepared by replacing Ni‐CB with CB.

### Electrocatalysts characterization

The crystalline structures of the prepared samples were analyzed by XRD (Bruker‐AXS Micro‐diffractometer D8 ADVANCE) equipped with a Cu_Kα_ radiation source (*λ*=1.54 Å) with a scan rate of 3° min^−1^. The crystallinity was further examined by Raman spectroscopy (Renishaw, with a 532 nm excitation laser). The samples were focused with a ×50LWD objective lens and exposed to emission line for 10 s.

TEM, HRTEM and EDX were conducted by JEM‐2100 Plus (JEOL) electron microscope operating at 200 kV. The HAADF‐STEM was conducted on JEOL NEOARM 200 F with 200 kV of accelerating voltage.

Nitrogen adsorption‐desorption isotherms were measured on Micromeritics Tristar II 3020 instrument at −196 °C. The specific surface area was estimated by the Brunauer–Emmett–Teller (BET) method, and the pore size distribution was obtained from the Barrett–Joyner–Halenda (BJH) desorption isotherm. The CO_2_‐TPD profile was measured on Micromeritics Autochem II ASAP 2920.

XPS was conducted on a 1486.6 eV X‐ray photoelectron spectrometer (ESCALAB Xi‐type) using Al_Kα_ source.

### Electrochemical measurements

All electrocatalytic tests were conducted by a standard three‐electrode system in an H‐type cell with two compartments at room temperature (25 °C). Platinum plate, catalysts coated carbon paper (Toray, TGP‐H060) and Ag/AgCl (3 m KCl) served as the counter electrode, working electrode and reference electrode, respectively. All potentials were controlled by AUTOLAB PGSTAT302N workstation and converted to the reversible hydrogen electrode (RHE) by *E* (vs. RHE)=*E* (vs. Ag/AgCl)+0.197 V+0.059×pH. The pH of 0.5 m KHCO_3_ saturated with CO_2_ is 7.2. Each chamber contained 40 ml 0.5 m KHCO_3_. The catalysts ink was prepared by sonicating a mixture of 3.0 mg catalysts, 145 μL ethanol, 90 μL DI water and 65 μL 0.5 wt.% Nafion‐D521 solution for 1 h. The working electrode was prepared by loading 60 μL catalysts ink onto a carbon paper (1 cm^2^) with a mass loading of 0.6 mg cm^−2^, followed by drying under an infrared lamp for 30 min. A piece of Nafion‐117 ionic exchange membrane was used to separate the anode chamber (counter electrode) and cathode (reference and working electrodes). High purity CO_2_ was pumped into the cathode chamber at a flow rate of 20 mL min^−1^ for 1 h before and during the electrolysis test. The 0.5 m KHCO_3_ electrolyte in the cathode was stirred at 800 rmp during the test. LSV was firstly performed under N_2_ and CO_2_‐saturated 0.5 m KHCO_3_ electrolyte at a scan rate of 10 mV s^−1^. The electrochemical double‐layer capacitance (*C*_dl_) was evaluated by the cyclic voltammetry (CV) method to estimate ECSA of the catalysts. The CV tests were conducted in 0.5 m KHCO_3_ electrolyte with a potential from 0 to −0.20 V (vs. Ag/AgCl) and various scan rates (10, 20, 40, 60 and 80 mV s^−1^) to avoid the faradaic process. *C*_dl_ can be obtained by plotting the anodic and cathodic current difference at −0.10 V against the scan rate. The ECSA can be determined by Equation (1)^[56]^ below:

(1) $$\mathrm{ECSA}=\frac{C_{dl}}{C_{s}}$$

where *C*_dl_ is the slope of the double‐layer charging current against the scan rate, and the value of *C*_s_ was chosen to be 40 μF cm^−2^ cm^−2^_ECSA_.

The gaseous products were analyzed by on‐line gas chromatography (Agilent GC‐7890B) equipped with a HayeSep Q column and a 5 A molecular sieve column. The possible liquid products were determined by nuclear magnetic resonance (NMR) spectroscopy. The NMR spectra were recorded on a 400 MHz Bruker NMR spectrometer in deuterium oxide (D_2_O) with one drop of dimethyl sulfoxide. ^1^H NMR chemical shifts were recorded in D_2_O. The FE of gaseous products (H_2_ and CO) under different potentials were calculated according to Equation 2:^[23]^


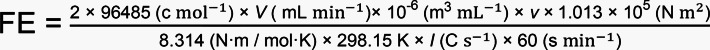




where V, v and I represent the gas flow rate, volumetric concentration of CO or H_2_, and steady‐state cell current at different potentials, respectively.

## Computational methods

All spin‐polarized calculations, including structure relaxation, electronic structure and energy, were performed by Vienna ab initio simulation package (VASP) using the plane‐wave basis.[^57^, ^58^] The generalized gradient approximation (GGA) in the parametrization of Perdew–Burke–Ernzerhof (PBE) were employed to describe the electronic exchange and correction.[^59^, ^60^] Projector augmented wave (PAW) pseudopotential was used to treat the interaction of core and valence electron.^[61]^ The long‐range van der Waals (vdW) interactions was considered by incorporating empirical correction method (DFT‐D3).^[62]^ The kinetic cut‐off energy was set to 500 eV. k‐point sampling of 8×8×1 (structure relaxation) and 10×10×1 (electronic structure calculations) for all systems were tested to achieve convergence. The convergence criterion for energy and force was set to 1.0×10^−5^ eV and 1.0×10^−2^ eV Å^−1^, respectively.

The Gibbs free energy change (▵*G*) of intermediates in each step for ECR and HER were calculated by the computational hydrogen electrode (CHE) model:^[63]^

(3) $$\Delta G=\Delta E+\Delta E{}_{\mathrm{ZPE}}-T\Delta S$$

where Δ*E*, Δ*E*_ZPE_ and Δ*S* are the differences in total energy obtained from DFT calculations, zero‐point energy, and entropy between reactants and products, respectively. *T* is temperature (298.15 K).

Two gaseous products (CO and H_2_) were considered in our calculations. The adsorption and desorption of intermediates are described by Equations (4)–7:

(4) $${}^{*}+\mathrm{CO}{}_{2}+\left(H{}^{+}+e{}^{-}\right)\to {}^{*}\mathrm{COOH}$$

(5) $${}^{*}\mathrm{COOH}+\left(H{}^{+}+e{}^{-}\right)\to {}^{*}\mathrm{CO}+H{}_{2}O$$

(6) $${}^{*}\mathrm{CO}\to \mathrm{CO}+{}^{*}$$

(7) $${}^{*}+\left(H{}^{+}+e{}^{-}\right)\to {}^{*}H$$

where * indicates an adsorption site.

## Conflict of interest

The authors declare no conflict of interest.

1

## Supporting information

As a service to our authors and readers, this journal provides supporting information supplied by the authors. Such materials are peer reviewed and may be re‐organized for online delivery, but are not copy‐edited or typeset. Technical support issues arising from supporting information (other than missing files) should be addressed to the authors.

Supporting Information

## Data Availability

The data that support the findings of this study are available from the corresponding author upon reasonable request.
