# Supplementary material for: Sulfur‐Decorated Ni−N−C Catalyst for Electrocatalytic CO2 Reduction with Near 100 % CO Selectivity
Source: ChemSusChem. 2022 Sep 1;15(19):e202200870. doi: 10.1002/cssc.202200870 (PMC9804562; doi:10.1002/cssc.202200870)
Supplement: Supplementary file 1 — Supporting Information [file CSSC-15-0-s001.pdf]

ChemSusChem

Supporting Information

Sulfur-Decorated Ni–N–C Catalyst for Electrocatalytic CO_2 Reduction with Near 100 % CO Selectivity

Song Lu, Yang Zhang, Mohamed F. Mady, Obinna Egwu Eleri, Wakshum Mekonnen Tucho, Michal Mazur, Ang Li, Fengliu Lou, Minfen Gu,* and Zhixin Yu*© 2022 The Authors. ChemSusChem published by Wiley-VCH GmbH. This is an open access article under the terms of the Creative Commons Attribution License, which permits use, distribution and reproduction in any medium, provided the original work is properly cited.

Figure S1. ^{1}H NMR of the possible liquid products after electrolysis for 1 h over Ni−NS−C catalyst at −0.80 V (vs. RHE) in CO_2-saturated 0.5 M $KHCO_3$.

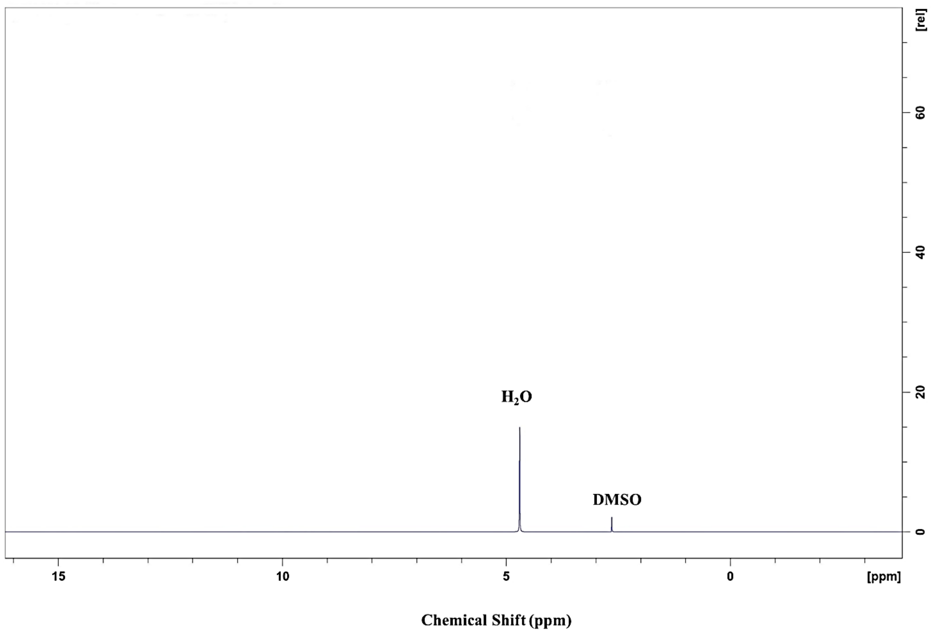

Figure S2. Cyclic voltammetry of (a) Ni−NS−C, (b) Ni−N−C, (c) NS−C, (d) N−C catalysts in CO_2-saturated 0.5 M $KHCO_3$ at various scan rates of 10, 20, 40, 60 and 80 mV/s; Cycle voltammetry was carried out between 0 and −0.2 V vs. Ag/AgCl.

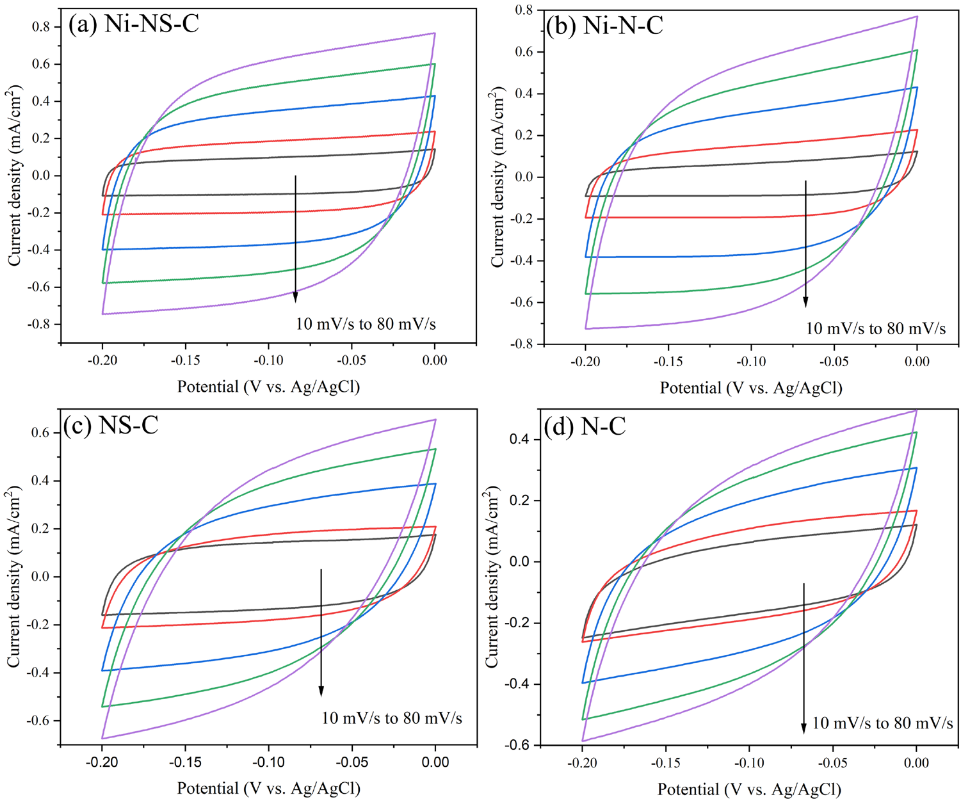

Figure S3. CO_2-TPD profiles of (a) Ni−NS−C and (b) Ni−N−C catalysts.

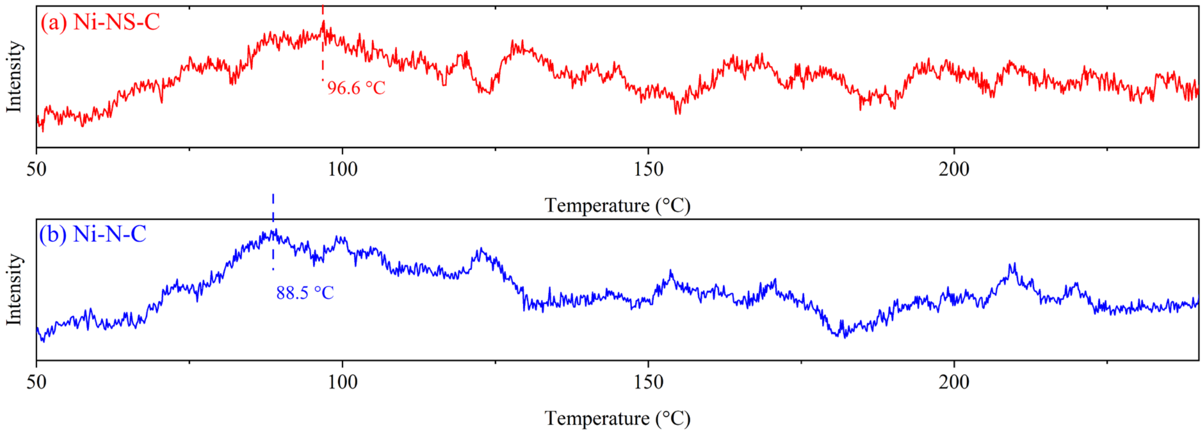

Figure S4. (a)-(c) TEM images and (d)-(g) EDX mapping of the spent Ni−NS−C catalyst.

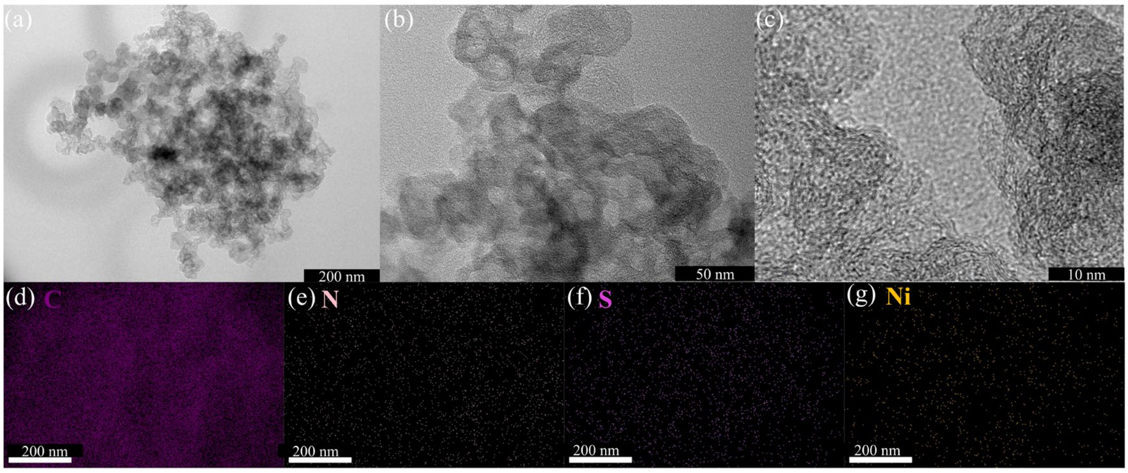

Figure S5. High-resolution XPS spectra (a) Ni 2p and (b) S 2p of the spent Ni−NS−C catalyst.

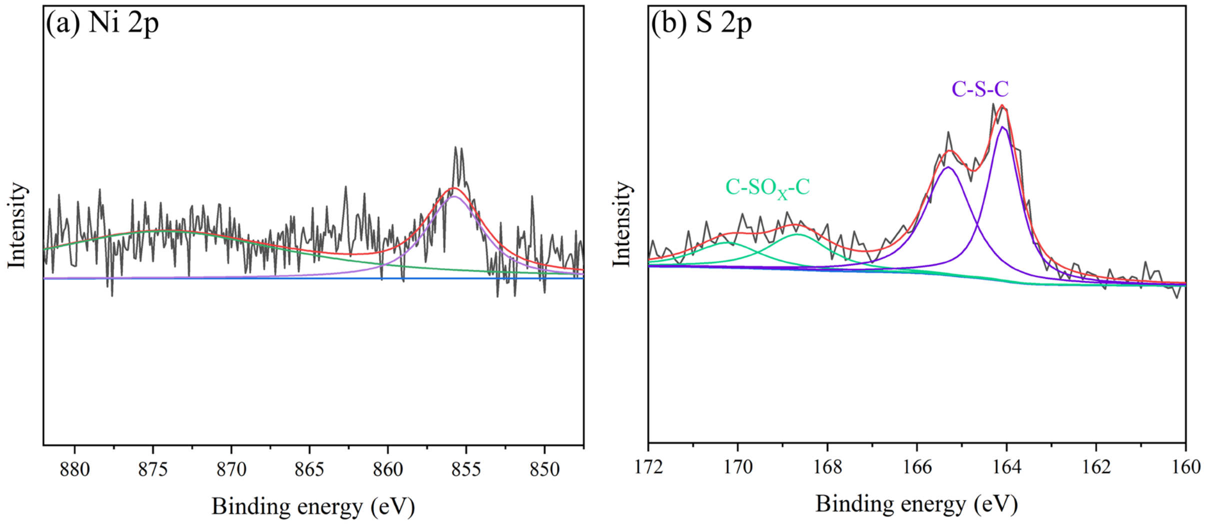

Figure S6. (a) A hypothetical structure, of which S substituted C atom without breaking TM−N_4; (b) free energy change of ECR to CO, and (c) free energy change of HER on the proposed structure.

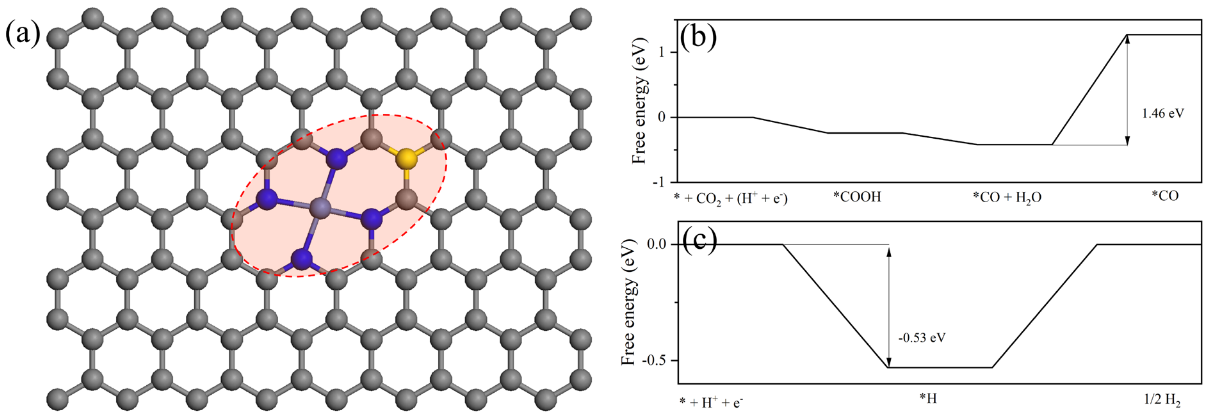

Table S1. Surface area and pore volume from N_2 adsorption-desorption study; Surface element contents from XPS measurement; ECSA from measuring double layer (D−L) capacitance.

Parameters	N−C	NS−C	Ni−N−C	Ni−NS−C	Ni−NS−C(spent)
Surface area (m^2/g)	1073	1120	1200	1275	−
Pore volume (m^3/g)	1.26	1.39	1.58	1.64	−
S (at. %)	−	0.37	−	0.42	0.40
N (at. %)	4.43	4.19	4.11	4.18	4.20
Ni (at. %)	−	−	0.50	0.48	0.45
ECSA (cm^2)	250	377	540	625	−

Table S2. Comparison of ECR performance for CO production on our Ni-NS-C catalyst and catalysts reported in literature.

Catalysts	FE (CO)	Potential vs. RHE	Stability	Reference
Ni−NS−C	99.7%	−0.80	19 h	This work
Ni/Fe−N−C	98%	−0.70	30 h	*Angew. Chem. Int. Ed*. 2019, 58, 6972
Ni_{SA}−N−C	71.9%	−0.90	60 h	*J. Am. Chem. Soc*. 2017, 139, 24, 8078
NiSA/GO	95%	−0.80	20 h	*Energy Environ. Sci*. 2018, 11, 893
Ni^{2+}@NG	92%	−0.68	20 h	*Adv. Mater*. 30 (2018) 1706617
C−Zn_1Ni_4	98%	−0.83	12 h	*Energy Environ. Sci*. 2018, 11, 1204
Ni−NCB	99%	−0.68	20 h	*Joule* 2019, 3, 265-278
Ni−N−C	94.8%	−0.86	29 h	*Chem. Eng. J* 2021, 131965
NiPc/NC	98%	−0.50	7 h	*ACS Sustainable Chem. Eng.* 2020, 8, 28, 10536
Ni−N_4	93%	−0.90	10 h	*Small* 2020, 16, 2003943
S−N−Ni/ACP	91%	−0.77	14 h	*J. Mater. Chem. A*, 2021,9, 1583
SA−Ni@NC	86.2%	−0.60	10 h	*Electrochem. commun* 2020, 116, 106758
Ni−SAs@FNC	95%	−0.77	10 h	*Appl. Catal. B: Environ* 2021, 283, 119591
Fe−NS−C	98%	−0.58	30 h	*Nano Energy* 2020, 68, 104384
FeSA−S/N−C	96.3	−0.49	24 h	*J CO_2 UTIL* 2020, 42 101316
(Cl, N)−Mn/G	97%	−0.60	12 h	*Nat Commun* 2019, 10, 2980
